# Dr. John J. Walsh

**Published:** 1905-12

**Authors:** 


Dr. John J. Walsh, of Buffalo, died at his home, 444 Porter Avenue, December 6, 1905, aged 57 years. He was born in Ireland, and when but a year old his parents removed to the United States, settling in Buffalo. His father, Dr. Nicholas Walsh, was at one time coroner of Erie County.

When a young man John J. Walsh entered upon the study of law and for a time served as law librarian at the city and county hall. Later, however, he turned his attention to medicine and graduated from the Medical Department of the University of Buffalo in 1871. He served as postmortem examiner and again was attached to the health department as sanitary inspector. He was also active in the National Guard, having served eleven years in the 65th and 74th regiments, and also was a member of Company D of the old Buffalo City Guard.

Dr. Walsh acquired a lucrative practice and gave up all public and military service in order to devote his entire time to his professional work. He became a member of the City, County and State Medical Societies, and was medical examiner for several benevolent associations. He is survived by a son, Attorney Walter B. Walsh; two brothers, Edward F. and Louis C. Walsh; and one sister, Mary J. Walsh. His wife died some years ago.

